# Predicting general and cancer-related distress in women with newly diagnosed breast cancer

**DOI:** 10.1186/s12885-016-2964-z

**Published:** 2016-12-03

**Authors:** Andrea Gibbons, AnnMarie Groarke, Karl Sweeney

**Affiliations:** 1 Health Psychology Research Unit, Royal Holloway, University of London, Egham, Surrey TW20 0EX UK; 2 School of Psychology, National University of Ireland, Galway, Ireland; 3 BreastCheck, the National Screening Programme, Western Unit, Galway, Ireland

**Keywords:** Cancer, Oncology, Breast cancer, Illness perceptions, Coping, Distress, Anxiety, Depression

## Abstract

**Background:**

Psychological distress can impact medical outcomes such as recovery from surgery and experience of side effects during treatment. Identifying the factors that explain variability in distress would guide future interventions aimed at decreasing distress. Two factors that have been implicated in distress are illness perceptions and coping, and are part of the Self-Regulatory Model of Illness Behaviour (SRM). The model suggests that coping mediates the relationship between illness perceptions and distress. Despite this; very little research has assessed this relationship with cancer-related distress, and none have examined women with screen-detected breast cancer. This study is the first to examine the relative contribution of illness perceptions and coping on general and cancer-related distress in women with screen-detected breast cancer.

**Methods:**

Women recently diagnosed with breast cancer (*N* = 94) who had yet to receive treatment completed measures of illness perceptions (Revised Illness Perception Questionnaire), cancer-specific coping (Mental Adjustment to Cancer Scale), general anxiety and depression (Hospital Anxiety and Depression scale), and cancer-related distress.

**Results:**

Hierarchical regression analyses revealed that medical variables, illness perceptions and coping predicted 50% of the variance in depression, 42% in general anxiety, and 40% in cancer-related distress. Believing in more emotional causes to breast cancer (β = .22, *p* = .021), more illness identity (β = .25, *p* = .004), greater anxious preoccupation (β = .23, *p* = .030), and less fighting spirit (β = −.31, *p* = .001) predicted greater depression. Greater illness coherence predicted less cancer-related distress (β = −.20, *p* = .043). Greater anxious preoccupation also led to greater general anxiety (β = .44, *p* < .001) and cancer-related distress (β = .37, *p* = .001). Mediation analyses revealed that holding greater beliefs in a chronic timeline, more severe consequences, greater illness identity and less illness coherence increases cancer-specific distress (*ps* < .001) only if women were also more anxiously preoccupied with their diagnosis.

**Conclusions:**

Screening women for anxious preoccupation may help identify women with screen-detected breast cancer at risk of experiencing high levels of cancer-related distress; whilst illness perceptions and coping could be targeted for use in future interventions to reduce distress.

## Background

Population-based screening for breast cancer is available in many countries to lower mortality rates through early detection of the disease [1]. Women are being treated successfully and surviving longer [2], so issues relating to quality of life and adjustment are becoming increasingly important [3], especially for women who are diagnosed through these screening programmes. Psychological distress is a common response to breast cancer, with women reporting clinical levels of anxiety and depression [4, 5]. Although anxiety and depression has been shown to decrease over time [6], a minority experience ongoing difficulties [7], and a recent study highlighted that women with screen-detected disease report less reduction in distress post-diagnosis compared with women with symptomatic disease [8]. Women who are distressed may experience more difficulties post-treatment, highlighting the need for countries to implement policies for screening of psychological distress in cancer patients [9]. Given that psychological distress can impact recovery from surgery, the experience of symptoms during treatment, and immune functioning [10–12], identifying the psychosocial factors that explain variability in anxiety and depression is an important challenge.

The Self-Regulatory Model of Illness Behaviour (SRM) [13] provides a framework for understanding how individual differences arise. It asserts that perceptions of an illness can impact upon an individuals’ response to a health threat. For breast cancer, women’s perceptions of their diagnosis can guide their coping with their illness and ultimately outcomes such as anxiety and depression. There are several illness perception dimensions; how long an illness will last (timeline beliefs), the seriousness of the disease (consequences), the ability to cure or control the disease (cure/control), how much sense the disease makes to an individual (illness coherence), the perceived cause (e.g., environment, stress, hereditary), and how much they identify themselves as having the disease (identity). These illness perceptions have been consistently associated with psychological functioning and adjustment across a wide variety of illnesses [14, 15] including cancer [16, 17], rheumatoid arthritis [18] and diabetes [19]. While research examining illness perceptions and distress in breast cancer is limited, holding chronic timeline beliefs, severe consequences, negative emotional representations, and psychological causal beliefs predict greater anxiety and depression [20]. Illness coherence has not been linked with anxiety in breast cancer, but is related to negative mood in gynaecological cancer [21]. Coping is also linked to anxiety and depression in breast cancer [6, 22, 23]. Problem-focused coping such as fighting spirit and seeking social support are adaptive and reduce distress, whilst certain emotion-focused styles such as denial and behavioural disengagement are associated with greater anxiety and depression [6, 24, 25].

Despite the inter-relationship of illness perceptions and coping in the SRM, very few studies have examined their impact simultaneously in breast cancer, and none have assessed women with screen-detected disease. Two such studies indicate that illness perceptions are stronger predictors of psychological distress than coping in concurrent analyses [26, 27]. In contrast, McCorry et al. [28] found that although illness perceptions and coping contributed to greater anxiety and depression at diagnosis, the influence of illness perceptions decreased while the influence of coping increased 6 months post-diagnosis. Variation in findings may stem from methodological variability. The measures were completed at diagnosis [28], before surgery [27], or within 2 years post-diagnosis [26]. Two studies used generic coping measures; it has been argued that these measures tend to reveal weaker relationships between illness perceptions and coping styles [29]. Likewise, it is suggested that within contextual models, appraisals, coping and emotional processes need to be assessed situationally [30].

The SRM asserts that coping mediates the relationship between illness perceptions and distress. Only one study has examined this in breast cancer and found no evidence of mediation [28]. Evidence with other illness groups is mixed; some report a mediational role for coping [21, 31] while others found no such evidence [14, 32]. A possible explanation for these contradictory findings is that illness perceptions and coping were examined for their ability to predict general rather than cancer-specific distress. It thus remains to be examined if illness perceptions and illness-related coping strategies explain more variability in cancer-specific distress than in general distress, and importantly to examine the mediational role of coping in relation to cancer-related distress in women with breast cancer.

The present study, therefore, compared for the first time, the effects of illness perceptions and illness-related coping on both general and cancer-specific distress in women recently diagnosed with breast cancer through a national screening programme. Specifically, it was hypothesized that holding beliefs of a strong illness identity, chronic timeline beliefs, severe consequences, low personal control, low levels of illness coherence, and a belief in psychological or emotional causes of breast cancer would predict greater anxiety, depression, and cancer-related distress. It was also hypothesised that coping would mediate the relationship between illness perceptions and cancer-specific distress.

## Methods

### Participants and procedure

Participants were recruited from a national breast cancer screening service in a large university affiliated hospital serving a large geographical area in Ireland. Consecutive women with a confirmed diagnosis of first-time breast cancer had not spread to local or distant metastases, who were 18 years of age or over, and able to read and write English were eligible to participate. Informed consent was obtained from the women in the clinic, after diagnosis but before commencement of their primary treatment. Participants completed the questionnaires and returned them to the principal investigator by post. Of the 334 eligible women approached, 289 (86.50%) agreed to take part, and of those, 94 (32.50%) returned the questionnaires.

### Materials and measures

Information on age, ethnicity, marital and employment status were collected. Type of surgery, stage of disease, type of diagnosis of breast cancer, and the type of treatment received (radiotherapy, chemotherapy, hormone therapy), were obtained from medical records.

### Predictors

Illness perceptions were measured using the Revised Illness Perception Questionnaire (IPQ-R) [33]. Women were asked to rate their agreement to statements about ‘my breast cancer’. The questionnaire yields a total of nine subscales, 6 of which were used in the current study: chronic timeline (e.g., my breast cancer will last for a long time; 6 items; α = .88), consequences (e.g., my breast cancer is a serious condition; 6 items; α = .75), personal control (e.g., there is a lot I can do to control my symptoms; 6 items; α = .77), illness coherence (e.g., my breast cancer doesn’t make sense to me; 5 items; α = .81), identity, and causes. All the items are rated on five point Likert scales ranging from 1 (*strongly disagree*) to 5 (*strongly agree*), except for those in the identity dimension. The identity subscale asks respondents to indicate from a list of 19 symptoms, whether they believe they are symptoms of breast cancer. Examples of symptoms include weight loss, fatigue, and pain, and the subscale has acceptable reliability (Cronbach’s α = .72). The causal items were used to calculate an emotional causes subscale (e.g., stress or worry, family problems; 6 items; α = .84), as previously identified in women with breast cancer [28]. Emotional representations were not included as they tend to correlate highly with anxiety and depression [14], and cyclical timeline and treatment control were excluded as they have not been indicated previously as a predictor of anxiety and depression in women with breast cancer. Greater scores on all subscales indicate stronger beliefs, so for example; higher consequences scores indicate greater perceived negative consequences, whilst high personal control scores indicated greater perceived personal control over breast cancer.

The fighting spirit and anxious preoccupation subscales of the Mental Adjustment to Cancer Scale (MAC) [34] were used to assess coping with breast cancer. Fighting spirit has 17 items and refers to an active coping style, for example “I have been doing what I believe will improve my health e.g., exercising”. Anxious preoccupation has 9 items and refers a more passive style of coping for example “I have difficulty in believing that this happened to me”. The other subscales (avoidance, fatalistic coping and helplessness/hopelessness) were not included as the number of predictors was limited to maximise power in the study, and it was hypothesised that fighting spirit and anxious preoccupation would be the main coping predictors of distress. Each item is rated on a four-point scale from 1 (*definitely does not apply to me*) to 4 (*definitely does apply to me*). Higher scores indicate higher levels of the coping style. Reliability scores of .79 were seen for fighting spirit, and .62 for anxious preoccupation.

### Outcomes

The Hospital Anxiety and Depression Scale (HADS) [35] was used to measure anxiety and depression. It is a 14 item scale (7 items for anxiety, 7 for depression) that asks individuals to indicate their level of agreement with statements on a four point scale from 0 to 3. Scores range from 0 to 21, for both scales, with higher scores indicating greater levels of anxiety or depression. Reliabilities in the current study ranged were .85 for depression, and .88 for anxiety.

Cancer-related distress was assessed by a series of questions adapted from previous research on cancer-specific distress [36, 37]. These items were used as other measures focus on the experience of symptoms of cancer rather than distress [38]. Participants were asked to rate how anxious, fearful, concerned, and worried they were about their diagnosis of breast cancer, from 1 (*not at all*) to 5 (*extremely*). Scores were summed to give a total cancer-related distress score. Scores range from 4 to 20, with higher scores indicating greater cancer-related distress. Internal reliability for the current sample was α = .96.

### Statistical analysis

SPSS 21 was used to conduct analyses. An Independent Samples *t* test was conducted to assess differences in age in women who did and did not respond. Chi Squared analyses were conducted to examine differences in stage of disease, type of surgery, and type of treatment in responders and non-responders. Pearson Product Moment correlations were conducted to examine the relationships between the predictors and outcome variables, as well as to identify medical and demographic factors to control for in the regressions. Hierarchical multiple regressions were conducted to examine the influence of illness perceptions and coping on cancer-related distress, anxiety, and depression in women with breast cancer. Mediation analyses were conducted using PROCESS [39] to assess whether there are any indirect effects of illness perceptions on cancer-specific distress, through coping. Using G*Power software [40] 76% power was achieved for the regression analyses.

## Results

### Study sample

All women were White; and the majority of women were married (80.80%). The remainder were separated or divorced (9.00%), single (3.80%), or widowed (6.40%). The majority of the sample was working (45.50%); others were working in the home (21.80%), retired (23.60%) or unemployed (9.10%). Most women (90.40%) underwent breast conserving surgery, with only 9.60% of women requiring a mastectomy (see Table 1). Most women received radiotherapy (80.90%) as part of their treatment.

**Table 1:** Descriptive statistics for study variables

Variable	Range	*M*	*SD*
Age	50–66	57.19	4.40
Illness perceptions (IPQ-R)			
Identity	0–9	2.25	2.29
Chronic Timeline	6–25	12.74	4.84
Consequences	9–30	19.67	4.81
Personal Control	10–30	21.96	4.39
Illness Coherence	8–25	17.53	4.42
Emotional Causes	6–27	14.36	4.65
Cancer-specific coping (MAC)			
Fighting Spirit	31–58	46.00	5.28
Anxious Preoccupation	12–33	21.96	3.86
Outcome variables			
Cancer-related distress	4–20	14.42	4.10
Anxiety (HADS)	0–19	7.97	4.48
Depression (HADS)	0–14	4.05	3.72
Demographics	%		
Marital status			
Single	3.80		
Married	80.80		
Separated/divorced	9.00		
Widowed	6.40		
Employment status			
Employed	45.50		
Working in the home	21.80		
Retired	23.80		
Unemployed	9.10		
Disease and treatment			
Stage of disease			
0	12.80		
IA, IB	46.80		
IIA, IIB	24.50		
IIIA, IIIB, IIIC	9.50		
Unspecified	6.40		
Treatment			
Mastectomy	9.60		
Excision	90.40		
Radiotherapy	80.90		
Chemotherapy	29.80		
Endocrine/hormone	54.30		

*Note*: *IPQ-R* Revised Illness Perception Questionnaire, *MAC* Mental Adjustment to Cancer Scale; *HADS* Hospital Anxiety and Depression Scale. Participants may have received both chemotherapy and radiotherapy

Using the cut-off scores adopted in previous research for identifying clinical levels of anxiety and depression [41], twenty eight women (30.40%) reported clinical levels of anxiety, and six women (6.50%) reported clinical depression scores. The mean score of cancer-related distress was 14.42 (*SD* = 4.10), with more than one third scoring 16 or above (37.20%), which indicates a high level of cancer-related distress.

There were no differences in age between those women who did and did not participate (*t*_(347)_ = −1.59, *p* = .112). Non-responders were more likely to have received a mastectomy than responders (*χ* ^2^ = 28.22, *df* = 4, *p* < .001). In addition, non-responders were more likely to have invasive cancer than -responders (*χ* ^2^ = 12.30, *df* = 4, *p* = .015). There were no differences in stage of disease (*χ* ^2^ = 6.68, *df* = 4, *p* = .154).

### Predictors of anxiety, depression, and cancer-related distress

Pearson Product moment correlations were conducted with the predictors and outcome variables (Table 2). Greater cancer-related distress was related to greater illness identity (*r* = .30, *p* = .007), a more chronic timeline (*r* = .38, *p* < .001), more severe consequences (*r* = .49, *p* < .001), less illness coherence (*r* = −.42, *p* < .001), and more use of anxious preoccupation as a coping strategy (*r* = .58, *p* < .001). Fighting spirt was negatively correlated with depression (*r* = −.32, *p* = .003), but not anxiety (*r* = −.14, *p* = .183), or cancer-specific distress (*r* = −.16, *p* = .140).

**Table 2:** Summary of intercorrelations between predictors and outcome variables

	1	2	3	4	5	6	7	8	9	10
1. Cancer Distress	-									
2. Anxiety	.66***	-								
3. Depression	.59***	.71***	-							
4. Identity	.30*	.25*	.40***	-						
5. Chronic Timeline	.38***	.42***	.41***	.14	-					
6. Consequences	.49***	.37**	.40***	.26**	.24**	-				
7. Personal Control	-.09	-.22*	-.12	-.14	.34***	-.10	-			
8. Illness Coherence	-.42***	-.39***	-.37***	-.02	-.26**	-.16	.08	-		
9. Emotional Causes	.19	.37***	.37***	.01	.22*	.11	-.21*	-.37***	-	
10. Fighting Spirit	-.16	-.14	-.32**	-.00	-.49***	-.04	.36***	.02	-.08	-
11. Anxious Preoccupation	.58***	.59***	.49***	.30**	.26*	.50***	.03	-.40***	.28**	.11

*p < .05, **p < .01, ***p < .001

Hierarchical multiple regressions were conducted to determine the influence of illness perceptions and coping, on cancer-related distress, and general distress (anxiety, and depression). Type of surgery, stage of disease, and type of cancer were controlled for in the first step. The order of the other variables in the regression were determined using the SRM model; which asserts that illness perceptions contribute to coping, which then determine adjustment such as distress. For this reason, identity, chronic timeline, consequences, personal control, illness coherence, and emotional causes were entered in the second step. Fighting spirit and anxious preoccupation were entered in the final step. Correlations between predictors ranged from .24 to .50 (see Table 2), and VIF scores ranged from 1.10 to 1.83 (tolerance scores ranged from 0.55 to 0.91), indicating that multicollinearity was not present.

As can be seen in Table 3, all of the models were significant. The medical variables did not predict variance in any of the outcomes. Illness perceptions accounted for 32% of the overall variance in cancer-related distress, 32% in anxiety, and 40% in depression. Greater illness coherence predicted lower cancer-related distress. A stronger illness identity and a greater belief in emotional causes of breast cancer predicted 40% of the variance in depression. Coping explained 10% of the variance in anxiety, 8% in depression, and 7% of cancer-related distress. Lower levels of fighting spirit and higher levels of anxious preoccupation predicted greater depression. Greater anxious preoccupation was also related to greater anxiety and cancer-related distress.

**Table 3:** Hierarchical multiple regressions explaining depression, anxiety, and cancer-related distress (*N* = 105)

Predictors	Depression (HADS)			Anxiety (HADS)			Cancer-related distress		
	β	*F* ^change^	*Adj. R* ^2^ ^change^	β	*F* ^change^	*Adj. R* ^2^ ^change^	β	*F* ^change^	*Adj. R* ^change^
(1) Medical variables		1.45	.02		0.66	.00		1.39	.01
Type of Surgery	.18*			.08			.16		
Diagnosis	-.09			.02			-.03		
Stage of disease	.05			-.01			-.02		
(2) Illness Perceptions		11.07***	.40		7.76***	.32		9.40***	.32
Identity	.25***			.07			.09		
Chronic Timeline	.15			.19			.13		
Consequences	.12			-.01			.15		
Personal control	.12			-.11			.04		
Illness coherence	-.11			-.06			-.20*		
Emotional Causes	.22*			.17			-.01		
(3) Coping		6.86**	.08		8.20***	.10		5.94***	.07
Fighting Spirit	-.31***			-.05			-.17		
Anxious Preoccupation	.23*			.44***			.37***		
Total *Adj R*2			.50			.42			.40

*Note.* *HADS* Hospital Anxiety and Depression Scale. *p < .05, **p < .01, ***p < .005. The variance explained by each group of variables, and the overall variance explained, can be found in the *Adj. R* ^*2*^ change column for each outcome. For example, illness perceptions accounted for 40% of the variance in depression

### Mediation analyses

To assess whether coping mediated the relationship between illness perceptions and cancer-related distress, analyses were conducted using PROCESS software [39]. It is an additional macro for SPSS that can estimate direct and indirect effects in singular mediator models using an ordinary least squares regression model [42]. These analyses were conducted to assess the direct and indirect effects of illness perceptions (identity, chronic timeline, consequences and illness coherence) on cancer-related distress, assessing anxious preoccupation as a potential mediator. Fighting spirit was not assessed as a potential mediator as it was not correlated with cancer-specific distress. Due to the small sample size, and to control for non-normal sampling distribution of the indirect effect, bootstrapping was included in the analyses, using an iteration of 5000, in line with recommendations [43]. The illness perceptions were assessed separately, but all analyses can be seen in Table 4.

**Table 4:** Unstandardized OLS regression coefficients with confidence intervals estimating anxious preoccupation and cancer-related distress

		Anxious preoccupation (M)			Cancer-related distress (Y)	
		Coefficient	95% CI		Coefficient	95% CI
Illness coherence (X)	*a1*	-.33***	(−0.41, −0.25)	*c1*	-.20***	(−0.28, −0.12)
Anxious preoccupation (M)				*b1*	.49***	(0.40, 0.59)
Indirect effect				*a1xb1*	-.16***	(−0.27, −0.09)
Constant		27.91***	(26.41, 29.41)		7.07***	(4.09, 10.04)
		*R*2 = .15, F _(1, 355)_ = 61.98, *p* < .001			*R*2 = .35, F _(2, 354)_ = 96.24, *p* < .001	
Chronic timeline (X)	*a2*	.20***	(0.12, 0.28)	*c2*	.20***	(0.13, 0.27)
Anxious preoccupation (M)				*b2*	.52***	(0.43, 0.61)
Indirect effect				*a2xb2*	.10***	(0.02, 0.20)
Constant		19.44***	(18.42, 20.46)		0.43	(−1.52, 2.38)
		*R*2 = .07, F _(1, 355)_ = 24.59, *p* < .001			*R*2 = .37, F _(2, 354)_ = 101.62, *p* < .001	
Consequences (X)	*a3*	.40***	(0.32, 0.47)	*c3*	.20***	(0.12, 0.28)
Anxious preoccupation (M)				*b3*	.46***	(0.35, 0.56)
Indirect effect				*a3xb3*	.18***	(0.10, 0.29)
Constant		14.17***	(12.72, 15.61)		0.29	(−1.70, 2.28)
		*R*2 = .24, F _(1, 355)_ = 114.47, *p* < .001			*R*2 = .35, F _(2, 354)_ = 96.76, *p* < .001	
Identity (X)	*a4*	.49***	(0.32, 0.66)	*c4*	.22*	(0.06, 0.38)
Anxious preoccupation (M)				*b4*	.55***	(0.45, 0.64)
Indirect effect				*a4xb4*	.27***	(0.09, 0.48)
Constant	20.87***	(20.44, 21.31)			1.83	(−0.15, 3.81)
		*R*2 = .08, F _(1, 355)_ = 31.24, *p* < .001			*R*2 = .33, F _(2, 354)_ = 85.08, *p* < .001	

*p < .05, **p < .01, ***p < .005

**Fig. 1:**
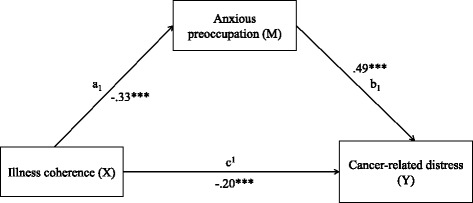
Conceptual model of effect of illness coherence and anxious preoccupation on cancer-related distress, with coefficients. M = mediator; X = dependent variable; Y = outcome variable; a_1_ = direct effect of illness coherence on anxious preoccupation; b_1_ = direct effect of anxious preoccupation on cancer-related distress; c_1_ = direct effect of illness coherence on cancer-related distress

As can be seen in Table 4 and Fig. 1, illness coherence had both a direct (*c* ^*1*^ = −.20***, CI = −0.28 to −0.12, *p* < .001) and indirect effect (*a*_*1*_ *xb*_*1*_ = −.16***, CI = −0.27 to −0.09, *p* < .001) on cancer-related distress. Anxious preoccupation also had a strong direct effect on cancer-related distress (*b*_*1*_ = .49***, CI = 0.40 to 0.59, *p* < .001). There were also direct and indirect effects of chronic timeline (*c* ^*2*^ = .20***, CI = 0.13 to 0.27, *p* < .001; *a*_*2*_ *xb*_*2*_ = .10***, CI = 0.02 to 0.20, *p* < .001), consequences (*c* ^*3*^ = .20***, CI = 0.12 to 0.28, *p* < .001; *a*_*3*_ *xb*_*3*_ = .18***, CI = 0.10 to 0.29, *p* < .001), and identity (*c* ^*4*^ = .22*, CI = 0.06 to 0.38, *p* < .001; *a*_*4*_ *xb*_*4*_ = .27***, CI = 0.09 to 0.48, *p* < .001), on cancer-related distress. Overall these results indicate that anxious preoccupation mediates the relationship between illness perceptions and cancer-related distress.

## Discussion

In this study, medical variables did not predict anxiety, depression, or cancer-specific distress. This is in contrast to previous research [44], but individuals do not always have adequate knowledge of the medical indices of their disease [45, 46], and hence these variables would not then necessarily predict psychological adjustment.

Overall, illness perceptions predicted a third or more of the variance in general and cancer-specific distress in women with screen-detected breast cancer. Specifically, higher levels of identity predicted greater levels of depression. Identity has been consistently shown to predict adjustment in patients with various forms of cancer, including breast cancer [27, 28, 47], and has been reported as an important predictor of anxiety in a recent meta-analysis [14]. These findings suggest that interventions which address symptom appraisal and symptom management may be useful in regulating anxiety and depression at diagnosis.

Illness coherence was the only illness perception to predict cancer-related distress, but it accounted for 32% of the variance. Women with newly diagnosed breast cancer may feel less distressed about their breast cancer if they have a clear sense of the disease and a greater understanding of it. Illness coherence may overlap with perceived knowledge and studies have shown that perceived receipt of more disease-specific information [48] and higher satisfaction with such information [49] are related to better understanding of illness in cancer patients. Further research examining the relationships between information provision, illness coherence and cancer distress is needed. Current findings; however, do suggest that strategies to enhance illness coherence at diagnosis may be useful. For example, provision of early stage health education information with clear explanations, may have a role in alleviating cancer distress.

Greater perceived control has typically predicted less anxiety and depression in breast cancer [27, 28], and control has been noted as one of the strongest of the illness perceptions as predictors of depression [14]. It may be that perceived personal control is less important for women who have screen-detected disease, as their prognosis is good and the majority women do not require invasive treatment. Beliefs in emotional causes for example stress or worry, predicted greater depression, but not cancer-specific distress. This may link to the controllability of risk where a person may be more anxious if they are unable to control or modify their exposure to a risk (e.g., stress, family problems). Stress is often considered a cause of breast cancer [50, 51], and can indeed predict health behaviours after a cancer diagnosis [52] as well as anxiety and depression [19]. Further research examining the role of causal attributions in distress as well as behaviour change will indicate how these may be included usefully in future interventions.

Results also support the relevance of coping to emotional adjustment in women with breast cancer. Higher levels of fighting spirit predicted less depression, whilst higher anxious preoccupation predicted greater cancer-related distress, anxiety, and depression. This is in line with the established literature in breast cancer that contends that active coping styles are adaptive, whilst passive or emotion-focused styles such as anxious preoccupation are maladaptive [6, 19]. Women, therefore, who ruminate anxiously on their illness at diagnosis, are at higher risk of both general and cancer-related distress so screening for this would allow for timely psychological support. The findings overall, suggest that illness perceptions outweigh the impact of illness-specific coping as predictors of both general and cancer-related distress in women with breast cancer. However, through anxious preoccupation coping, illness coherence can indirectly affect cancer-related distress. This fits with conclusions in a recent meta-analysis [14]; strategies such as avoidance and venting of emotions rather than positive coping styles mediate the relationship between illness perceptions and adjustment in illness. Modification of coping may, therefore, change the relationship between illness perceptions and cancer-related distress. Illness perceptions may be difficult to modify [28], whereas coping strategies may be more amenable to change. This is one of the few studies to demonstrate the presence of mediation [14, 21, 31], and suggests that reducing anxious negative rumination may help to influence the link between specific illness perceptions and cancer-related distress. Furthermore, this finding validates the SRM model and adds to the literature on the mediational role of negative coping in people with cancer. The differences across outcomes indicate that illness coherence is influential in cancer-related distress, whilst identity, personal control, and causal beliefs influence general anxiety and depression. This underscores the value of including assessment of both general and specific distress when measuring the impact of illness perceptions.

There are limitations to this study. The study was cross-sectional so causal inferences cannot be made. Despite this, it indicates that illness perceptions and coping are influential in distress at diagnosis. The sample had screen-detected disease, and non-responders were more likely to have more invasive disease, requiring more invasive treatment. The results therefore, are only generalisable to women who are diagnosed through screening. The emergence of standardised national screening programmes will reduce the number of self-detected cancers, however, as well as the stage of disease and percentage of invasive cancers, so results here are important for determining how this group responds to a cancer diagnosis. The study has a modest sample size. Recruitment of cancer patients is challenging, especially at diagnosis, and while the response rate for return of questionnaires was disappointing, they were consecutive women attending breast clinics with a confirmed diagnosis of breast cancer.

## Conclusions

Overall, the current study has important implications for adjustment in women with breast cancer. This is one of the few studies that included measures of illness perceptions and coping and it demonstrates their role in explaining variance in both cancer-related and general distress at diagnosis of breast cancer. The present study is also the first to confirm that illness-related coping mediates the relationship between illness perceptions and cancer-related distress in breast cancer. Although more work is warranted, it provides further insight into the relationship of these components within the Self-Regulatory model. By identifying determinants of general and cancer-related distress in women with breast cancer, these results will help to identify those at risk for poor adaptation and inform the design of psychological interventions to reduce distress, which may lead to improvements in medical outcomes.

## Abbreviations

HADS: Hospital anxiety and depression scale
IPQ-R: Revised illness perception questionnaire
MAC: Mental adjustment to cancer scale
SRM: Self-regulatory model
